# Antibody and T cell responses to COVID-19 vaccination in patients receiving anticancer therapies

**DOI:** 10.1136/jitc-2022-004766

**Published:** 2022-06-22

**Authors:** Sherin Juliet Rouhani, Jovian Yu, Daniel Olson, Yuanyuan Zha, Apameh Pezeshk, Alexandra Cabanov, Athalia R Pyzer, Jonathan Trujillo, Benjamin A Derman, Peter O'Donnell, Andrzej Jakubowiak, Hedy L Kindler, Christine Bestvina, Thomas F Gajewski

**Affiliations:** 1 Department of Medicine, University of Chicago, Chicago, Illinois, USA; 2 Department of Pathology, University of Chicago, Chicago, Illinois, USA

**Keywords:** COVID-19, immunogenicity, vaccine, immunotherapy, antibody formation, T-lymphocytes

## Abstract

**Background:**

Patients with cancer were excluded from phase 3 COVID-19 vaccine trials, and the immunogenicity and side effect profiles of these vaccines in this population is not well understood. Patients with cancer can be immunocompromised from chemotherapy, corticosteroids, or the cancer itself, which may affect cellular and/or humoral responses to vaccination. PD-1 is expressed on T effector cells, T follicular helper cells and B cells, leading us to hypothesize that anti-PD-1 immunotherapies may augment antibody or T cell generation after vaccination.

**Methods:**

Antibodies to the SARS-CoV-2 receptor binding domain (RBD) and spike protein were assessed in patients with cancer (n=118) and healthy donors (HD, n=22) after 1, 2 or 3 mRNA vaccine doses. CD4^+^ and CD8^+^ T cell reactivity to wild-type (WT) or B.1.617.2 (delta) spike peptides was measured by intracellular cytokine staining.

**Results:**

Oncology patients without prior COVID-19 infections receiving immunotherapy (n=36), chemotherapy (n=15), chemoimmunotherapy (n=6), endocrine or targeted therapies (n=6) and those not on active treatment (n=26) had similar RBD and Spike IgG antibody titers to HDs after two vaccinations. Contrary to our hypothesis, PD-1 blockade did not augment antibody titers or T cell responses. Patients receiving B-cell directed therapies (n=14) including anti-CD20 antibodies and multiple myeloma therapies had decreased antibody titers, and 9/14 of these patients were seronegative for RBD antibodies. No differences were observed in WT spike-reactive CD4^+^ and CD8^+^ T cell generation between treatment groups. 11/13 evaluable patients seronegative for RBD had a detectable WT spike-reactive CD4^+^ T cell response. T cells cross-reactive against the B.1.617.2 variant spike peptides were detected in 31/59 participants. Two patients with prior immune checkpoint inhibitor-related adrenal insufficiency had symptomatic hypoadrenalism after vaccination.

**Conclusions:**

COVID-19 vaccinations are safe and immunogenic in patients with solid tumors, who developed similar antibody and T cell responses compared with HDs. Patients on B-cell directed therapies may fail to generate RBD antibodies after vaccination and should be considered for prophylactic antibody treatments. Many seronegative patients do develop a T cell response, which may have an anti-viral effect. Patients with pre-existing adrenal insufficiency may need to take stress dose steroids during vaccination to avoid adrenal crisis.

**What is already known on this topic**

- The phase 3 trials investigating the efficacy of the COVID-19 vaccines excluded patients with cancer. Over the last year, a number of studies have measured the antibody response to COVID-19 vaccination in patients with cancer, but few studies have also investigated the T cell response.

**What this study adds**

- This study provides a comprehensive analysis of the antibody and T cell responses in patients with cancer after COVID-19 vaccination, and shows that many patients on B cell-targeted therapies do not develop detectable antibodies yet do develop detectable T cell responses.

**How this study might affect research, practice or policy**

- This study suggests that anti-receptor binding domain antibody levels should be routinely checked in selected patients with cancer after COVID-19 vaccination, and patients without detectable antibody levels should be prioritized for pre-exposure prophylactic antibody administration. Oncologists should be aware of the potential for vaccination to trigger an adrenal crisis, and patients with known adrenal insufficiency should be counseled to have a low-threshold to take stress-dose steroids if they develop a fever or signs of adrenal crisis postvaccination.

## Introduction

Several SARS-CoV-2 vaccines have been approved or received emergency use authorization, and offer significant hope toward mitigating the COVID-19 pandemic. However, the phase 3 studies leading to regulatory approval excluded immunocompromised patients,^1–3^ so the ability of patients receiving chemotherapy, immunotherapy, or other immunosuppressive treatments to mount comparable immune responses after vaccination needs further investigation. Cancer is a risk factor for severe COVID-19.^4 5^ Therefore, understanding the degree of protection that vaccination offers to this population is essential.

The spike protein is the antigenic target of both the mRNA-1273 (Moderna) and BNT162b2 (Pfizer BioNTech) mRNA vaccines as well as the Ad26.COV2.S (Johnson & Johnson Janssen) and ChAdOx1 nCoV-19 (Oxford-AstraZeneca) adenoviral vaccines. Neutralizing antibodies prevent infection by blocking the ability of the receptor binding domain (RBD) of the spike protein to bind to ACE2 and infect host cells. RBD is the predominant target of neutralizing antibodies,^6^ and RBD binding antibody levels strongly correlate with neutralization and/or pseudoneutralization titers,^7–10^ as well as protection against severe disease.^11^ Spike titers are highly correlated with RBD titers.^8^ Anti-spike reactive antibodies may also include non-neutralizing antibodies directed against other epitopes on the Spike protein that can aid viral recognition and clearance after infection. The antibody levels required for protection from infection are unknown, although several models have shown that binding and neutralizing antibodies normalized to levels in human convalescent sera robustly correlate with vaccine efficacy in phase 3 trials.^12 13^ These models suggest that 50% protective vaccine efficacy is achieved with approximately 10%–20% of the mean convalescent antibody levels.

Much of the work to date investigating the immune response to vaccination and its kinetics has been done in healthy donors (HDs). COVID-19 vaccines induce a Th1-polarized CD4^+^ response, with IFN-γ, TNF-α, and IL-2 production being observed by both antigen-specific CD4^+^ and CD8^+^ T cells.^8 9^ In HDs, both antibody and T cell responses are detectable for at least 6 months after vaccination.^10 14 15^ The half-life of RBD antibodies after infection or vaccination has ranged from 28 to 116 days^6^ ^10 14 16 17^ while the half-life for CD4^+^ and CD8^+^ T cells ranged from 47–207 days to 27–196 days, respectively.^10 16 17^ Booster vaccinations are recommended to enhance antibody and cellular responses, which predictably decline over time. In addition to augmenting antibody titers, the booster dose also enhances neutralization ability against novel variants, including delta and omicron.^18^

Patients with cancer can be immunocompromised after bone marrow suppression from current or prior chemotherapy, adjunctive medications such as corticosteroids, B-cell targeted therapies, or the cancer itself. Thus, it is important to evaluate the antibody and T cell responses to COVID-19 vaccines in patients with cancer receiving potentially immunosuppressive therapies. In addition, COVID-19 vaccine immunogenicity and safety data in patients with cancer receiving therapies that stimulate the immune system, such as immune checkpoint inhibitors, are also critically needed. The effects of immune checkpoint inhibitor therapy on the immune response after vaccination in humans is not well characterized. COVID-19 vaccination of patients receiving immunotherapy offers an opportunity to understand how anti-PD-1 agents affect the generation and contraction of the human immune response to a defined vaccine antigen in vivo. T cells upregulate PD-1 on activation, which inhibits effector function and affects memory generation.^19 20^ In murine tumor vaccine models where there is suboptimal priming of T cells in the tumor microenvironment prior to anti-tumor vaccination, treatment with a PD-1 inhibitor can lead to dysfunctional T cells after vaccination.^21^ However, this effect is not seen under optimal antigen priming conditions, such as those expected during COVID-19 vaccination. PD-1 and PD-L1 are also expressed by B cells, T follicular helper (T_FH_) and T follicular regulatory (T_FR_) cells. Blockade of the PD-1 pathway increases B cell activation and proliferation,^22 23^ but can also inhibit germinal center B cell survival.^24 25^ Signaling from T_FH_ cells promotes germinal center formation and is important for the development of high affinity antibodies and long-lived plasma cells.^26^ PD-L1^hi^ B regulatory cells suppress T_FH_ activation and proliferation, leading to decreased antibody generation.^27 28^ We hypothesized that patients receiving anti-PD-1 or PD-L1 antibodies might generate enhanced humoral and cellular immune responses compared with healthy controls, while patients on B cell-directed therapy or chemotherapy might have impaired responses. Understanding the T cell response to vaccination is critical as variants of concern evolve containing mutations that impair antibody neutralization, but less has been published thus far on the T cell response after vaccination. In this study, we examined the antibody and T cell responses to COVID-19 vaccination in oncology patients treated in a real world setting at the University of Chicago.

## Methods

### Study design and sample collection

Patients with cancer aged 18 years or older who were seen in the University of Chicago outpatient hematology/oncology clinics were approached to participate in the study. Blood samples were collected during a routine clinic or laboratory visit after the first, second, and/or third doses of the vaccine. These patients were categorized based on the type of cancer and type of treatment received at the time of vaccine doses 1–2. ‘B cell targeted agents’ were subclassified into anti-CD20 depleting antibodies (n=4) such as rituximab, and other plasma or B cell directed treatments including proteasome inhibitors (n=10) such as carfilzomib, anti-CD38 antibodies (n=2) such as daratumumab, Bruton’s tyrosine kinase (BTK) inhibitors (n=1), and venetoclax (n=1). Patients who received chemotherapy along with B cell targeted therapies were only analyzed in the B cell targeted therapy group. Immunotherapy included anti-PD-1/PD-L1 inhibitors±anti-CTLA-4 inhibitors, and 2 patients on an investigational bispecific antibody linking CD3 with a cancer-specific antigen. Patients who received cytotoxic chemotherapy along with immunotherapy were analyzed separately as a ‘ChemoIO’ category. The endocrine/targeted therapy group included patients who were only on endocrine therapies (n=2) such as aromatase inhibitors or GnRH analogs, or on targeted therapies such as BRAF/MEK inhibitors (n=2), vascular endothelial growth factor receptor (VEGFR) inhibitors (n=1), CDK4/6 inhibitors (n=1), or IDH2 inhibitors (n=1). Patients not on treatment included those who were on observation after completing treatment (n=19, either after completing adjuvant or neoadjuvant treatment, or patients who stopped treatment after achieving a complete response or stable disease), as well as newly diagnosed patients who had not yet started anticancer therapy at the time of the second vaccine dose (n=9). Chemotherapy, endocrine therapies, and targeted therapies given between 30 days prior to dose 1 through dose 2 were included. Given the longer half-life of monoclonal antibodies, patients who received immunotherapy or B cell depleting antibodies between 90 days prior to dose 1 through dose 2 were included in these categories.

Healthy controls and patients with prior infections were used for comparisons. Healthy controls predominantly consisted of healthcare workers vaccinated between December 2020 and March 2021. No healthy control had a known SARS-CoV-2 infection prior to vaccination. Antibody levels from non-immunocompromised patients and patients with cancer who were unvaccinated and had a SARS-CoV-2 infection were used as a comparison; results from the non-immunocompromised/non-cancer infected patient cohort have previously been reported separately as end-point titers^29^ and were reanalyzed as midpoint titers in this study. Infected patients all had a clinical positive diagnostic SARS-CoV-2 PCR test; 98% of infections occurred between March and June 2020. One patient with cancer was included with a COVID-19 infection from May 2021. Patients who were treated with anti-CD20 agents, mycophenolate mofetil, or who had a history of solid organ transplant were excluded from the infected non-immunocompromised cohort.

### Time points

Dose 1 time points included samples drawn 10 days after initial vaccination through the day of second vaccination. Dose 2 time points included samples drawn 7 days after second vaccination through the day of the third vaccination. Dose 3 time points included samples drawn at least 7 days after the third vaccination.

### Sample collection

For serum, fresh blood was collected into a preservative-free vacutainer tube and allowed to clot for at least 30 min at room temperature before being centrifuged for 20 min at 1300 x g at room temperature. The serum was then stored at −80°C until analysis. For peripheral blood mononuclear cells (PBMCs), fresh blood was collected into heparinized vacutainer tubes and peripheral mononuclear cells were separated using LeucoSep (Greiner Bio-One) tubes. Cells were washed twice with phosphate buffered saline (PBS), counted and resuspended in freezing media containing 90% fetal bovine serum (FBS) and 10% DMSO at 5–20×10^6^/mL. Cells were frozen in liquid nitrogen until further analysis.

### Antibody response

The SARS-CoV-2 wild-type (WT) Spike and RBD protein expression constructs created by Florian Krammer’s laboratory^30^ were used to generate recombinant protein for an ELISA adapted from established protocols.^30^ Recombinant proteins were produced using a Chinese hamster ovary cell line expression system and purified using metal-chelate affinity chromatography. Protein integrity was confirmed via SDS-PAGE gel. Overnight, 96-well ELISA plates (Nunc MaxiSorp high protein-binding capacity plate; ThermoFisher) were coated at 4°C with 2 mg/mL of Spike or RBD protein suspended in PBS pH 7.4. Plates were blocked with 3% milk powder in PBS containing 0.1% Tween-20 for 1 hour at room temperature. Serum and plasma samples were heated at 56°C for 30 min to inactivate virus prior to use. Serial 1:3 dilutions of the samples were prepared in 1% milk in 0.1% PBS-Tween 20, and incubated in duplicate with the blocked plate for 2 hours at room temperature. After 3 washes in 0.05% PBS-Tween 20, an HRP-conjugated IgG secondary antibody (Goat anti-human IgG (H+L), Jackson ImmunoResearch) was diluted in 1% milk in 0.1% PBS-Tween-20, and added at 1:8000 for 1 hour at room temperature. Plates were washed 3 times with 0.1% PBS-Tween 20 before being developed with 3, 3’, 5, 5’-tetramethylbenzidine (TMB) substrate kit (ThermoFisher) at room temperature. The reaction was stopped after 15 min with 2M sulfuric acid. The optical density (OD) was read at 450 nm using a Synergy H4 plate reader (BioTek). The OD values for each sample were background subtracted. A positive control standard was prepared from plasma samples pooled from 6 COVID-19-infected patients, while plasma from uninfected, unvaccinated individuals were used as a negative control standard. Titers were calculated using midpoint extrapolation, and normalized to negative control serum drawn. Seropositivity was defined as three times the mean of negative controls. All samples were run in duplicate and the mean value of the duplicates was used.

### T cell responses

For intracellular cytokine staining, PBMCs were thawed and washed with T cell media (RPMI with 10% FBS, 2 mM L-glutamine, 1 mM sodium pyruvate, 50 uM 2-BME, 100 U/mL penicillin, 100 mg/mL streptomycin), counted and resuspended at 10E6 cells / mL. Cells were plated in 200 µL in a 96 well U-bottom plate to rest overnight. The following day, 100 µL of media was removed from each well and replaced with 100 µL containing GolgiPlug and GolgiStop (BD biosciences), 5 µL αCD28/CD49d FastImmune (BD Biosciences, Cat #347690), and peptide pools as indicated. Unstimulated wells received αCD28/CD49d without additional peptides. Overlapping pooled peptides at 0.6 nmol/mL from either the Spike protein of either the original Wuhan variant (PepTivator SARS-CoV-2 Prot_S, Miltenyi Biotec 130-126-700) or the B.1.617.2 variant (PepTivator SARS-CoV-2 Prot_S B.1.617.2, Miltenyi Biotec, 130-128-763) were used for spike peptide stimulated wells. The WT spike peptide pool consisted of 15-mer sequences with 11 amino acid overlap covering many of the predicted antigenic sequences (amino acids 304–338, 421–475, 492–519, 683–707, 741–770, 785–802, and 885–1273). In contrast, the B.1.617.2 spike peptide pool consists of 32 peptides of 15 amino acids in length, which selectively covers the 10 mutations that differ between the WT and B.1.617.2 lineage spike proteins (T19R, G142D, E156G, deletion 157, deletion 158, L452R, T478K, D614G, P681R, and D950N). The CEF PepTivator peptide pool (Miltenyi Biotec, 130-098-426) was used as a positive control. If <8E6 PBMCs were recovered, the unstimulated and WT spike-peptide wells were prioritized, followed by B.1.617.2 spike-peptide and CEF wells.

After a 9- hour incubation, cells were stained in a 96-well V-bottom plate. Cells were stained with live/dead blue (Invitrogen) in PBS for 15 min in 25 µL prior to adding monocyte blocker (Biolegend, Cat #426103, 5 µL), Fc Block (Biolegend Cat #422302, 5 µL), CCR7 BV421 (Clone G043H7, Biolegend, 5µL), and CXCR5 PE (J252D4, Biolegend, 5 µL) and 5 µL FACS buffer for 10 min. The remainder of the extracellular antibodies (CD3 AlexaFluor532 (UCHT1, ThermoFisher, 5 µL), PD-1 BV785 (EH12.2H7, Biolegend, 5 µL), CD4 AlexaFluor700 (SK3, Biolegend, 5 µL), CD19 SparkNIR685 (HIB19, Biolegend, 2 µL), CD8 BUV805 (SK1, BD, 1.2 µL), CD16 BUV496 (3G8, BD, 0.6 µL), CD45RA BUV395 (5H9, BD, 0.3 µL)) and 10 µL brilliant stain buffer plus (BD) were added for 30 min for a total volume of 100 µL. Cells were washed and were resuspended in the fixation/permeabilization solution from eBioscience’s Cyto-Fast Fix/perm kit (Biolegend, Cat #426803) for 30 min, washed, and incubated in 100 µL permeabilization buffer with intracellular antibodies (IFNy AF488 (4S.B3, Biolegend, 5 µL), TNFa BV605 (MAb11, Biolegend, 5 µL), IL-2 BV650 (MQ1-17H12, Biolegend, 5 µL) for 30 min. Cells were washed 2 x with permeabilization buffer prior to being resuspended in FACS buffer and run on the Cytek Aurora. Data analyzed using FlowJo V.10.

### Data analysis

Clinical data including cancer and treatment details as well as immune-related adverse events (irAEs) were abstracted from the electronic medical record by a clinical data manager or research coordinator. Patients were asked to verify their vaccine dates, if they were seen in an emergency room or hospitalized within 24 hours of the first or second dose of the vaccine, and if they had previously been diagnosed with COVID-19. Treatment categorization was verified by an oncologist (DO or SJR). Researchers were blinded to groups during conduct and analysis of experiments. Flow cytometry data were processed using FlowJo V.10.7.1. Graphs were created and statistics performed using either GraphPad Prism V.9.0.0 or R V.4.0.3 (R Core Team, 2020). R packages used include drc,^31^ ggpmisc,^32^ ggpubr,^33^ ggsignif,^34^ gridExtra,^35^ Hmisc,^36^janitor,^37^ lubridate,^38^ magrittr,^39^ patchwork,^40^ readxl,^41^ reshape2,^42^ scales,^43^ stringr,^44^ tidyverse,^45^ writexl.^46^ Full reproducible code for data processing is available at https://github.com/jovianyu/ucm_covid19_vaccine.

### Statistical analysis

Throughout the paper, boxplots show the medians (middle line) and the first and third quartiles (upper and lower bounds of the boxes). Significance of comparisons from boxplots were determined by two-sided Mann-Whitney Wilcoxon test and significance is expressed as p values, shown as asterisks (*p≤0.05; **p≤0.01; ***p≤0.001). All replicates shown are from distinct samples.

## Results

### Patient demographics and cohort

We collected serum and peripheral blood mononuclear cells (PBMCs) during standard clinical visits from 118 oncology patients and 22 HD who were vaccinated with at least 2 doses of either the mRNA-1273 (Moderna) or BNT162b2 (Pfizer BioNTech) mRNA COVID-19 vaccines. The HD cohort consisted predominantly of healthcare workers and employees vaccinated with the two-dose Pfizer vaccine between December 2020 and March 2021, while patient vaccinations occurred between December 2020 and June 2021. Participant demographics are shown in table 1. Our vaccinated oncology cohort was predominantly composed of patients with solid tumors (81%) vs hematological (19%) malignancies, and the most common tumor types represented were melanoma (25%), thoracic malignancies (20%), and gastrointestinal malignancies (14%). Seventeen per cent of patients were African-American and 77% were Caucasian. The average patient age was 63 years old (range 28–81). Antibody titers from unvaccinated patients who had a documented SARS-CoV-2 infection between March 2020 and May 2021 were used as a comparison. Samples from the infection cohort were taken an average of 25 days post-symptom onset (range 10–87 days). These infected patients were separated into patients with a history of cancer (solid tumor, n=6; hematological malignancies, n=4) and non-immunocompromised patients without a history of cancer (n=55).

**Table 1:** Participant characteristics

		Oncology patients (Vaccinated)							HD (vaccinated)	SARS-CoV-2 infection	
		Entire cancer cohort	IO	Chemo	Chemo+IO	B cell targeted agents	Targeted therapy or hormonal tx	Not on active tx		Non-IMCD, non-cancer	Cancer
		n=118	n=38	n=19	n=7	n=18	n=8	n=28	n=22	n=55	n=10
Age, mean in years (range)		63 (28–81)	66 (45–81)	59 (30–79)	60 (37–73)	65 (59–76)	63 (50–71)	61 (28–81)	48 (31–67)	56 (31->90)	62 (36–76)
Male sex, n (%)		56 (47%)	13 (34%)	9 (47%)	4 (50%)	10 (56%)	6 (75%)	14 (50%)	10 (45%)	31 (56%)	4 (40%)
Race, n (%)											
	African-American	20 (17%)	7 (18%)	6 (32%)	1 (14%)	2 (11%)	1 (13%)	3 (11%)	0 (0%)	41 (75%)	7 (70%)
	Caucasian	91 (77%)	28 (74%)	12 (63%)	6 (86%)	15 (83%)	6 (75%)	24 (86%)	15 (68%)	6 (11%)	2 (20%)
	Asian	2 (2%)	1 (3%)	0 (%)	0 (%)	1 (6%)	0 (0%)	0 (0%)	6 (27%)	1 (2%)	0 (0%)
	Did not disclose/more than 1	5 (5%)	2 (5%)	1 (5%)	0 (%)	0 (%)	1 (13%)	1 (3.6%)	1 (5%)	7 (13%)	1 (11%)
Vaccine manufacturer, n (%)											
	Pfizer	101 (86%)	30 (79%)	15 (79%)	7 (100%)	18 (100%)	8 (100%)	23 (82%)	22 (100%)	N/A	N/A
	Moderna	17 (14%)	8 (21%)	4 (21%)	0 (0%)	0 (0%)	0 (0%)	5 (18%)	0 (0%)	N/A	N/A
Cancer type, n (%)											
	Melanoma or skin	30 (25%)	11 (29%)	3 (16%)	0 (%)	1 (6%)	0 (0%)	15 (54%)	-	-	1 (10%)
	Thoracic	24 (20%)	11 (29%)	5 (26%)	4 (57%)	0 (%)	2 (25%)	2 (7%)	-	-	2 (20%)
	GI	17 (14%)	5 (13%)	9 (47%)	1 (14%)	0 (%)	0 (0%)	2 (7%)	-	-	0 (0%)
	GU	14 (12%)	5 (13%)	2 (11%)	1 (14%)	0 (%)	4 (50%)	2 (7%)	-	-	0 (0%)
	Head & neck, endocrine	5 (4%)	2 (5%)	0 (%)	1 (14%)	0 (%)	0 (0%)	2 (7%)	-	-	1 (10%)
	Breast/Gyn	6 (5%)	3 (8%)	0 (%)	0 (%)	0 (%)	1 (13%)	2 (7%)	-	-	2 (20%)
	Hematological malignancy	22 (19%)	1 (3%)	0 (%)	0 (%)	17 (94%)	1 (13%)	3 (11%)	-	-	4 (40%)
Stage, n (%)											
	I or II - on active tx	4 (3%)	2 (5%)	2 (11%)	0 (%)	0 (%)	0 (0%)	0 (0%)	-	-	-
	I or II - previously resected, currently NED	3 (3%)	0 (0%)	0 (%)	0 (%)	0 (%)	0 (0%)	3 (11%)	-	-	2 (20%)
	III - on active tx	11 (9%)	4 (11%)	4 (21%)	3 (43%)	0 (%)	0 (0%)	0 (0%)	-	-	1 (10%)
	III - previously resected, currently NED	6 (5%)	0 (0%)	0 (%)	0 (%)	0 (%)	1 (13%)	5 (18%)	-	-	-
	IV	71 (60%)	31 (82%)	13 (68%)	4 (57%)	0 (%)	6 (75)	17 (61%)	-	-	1 (10%)
	Non-TNM staging or NR	23 (20%)	1 (3%)	0 (%)	0 (%)	18 (100%)	1 (13%)	3 (11%)	-	-	6 (60%)
ECOG PS, n (%)											
	0	73 (62%)	16 (42%)	10 (53%)	3 (43%)	15 (83%)	7 (88%)	22 (79%)	-	-	3 (30%)
	1	37 (31%)	19 (50%)	7 (37%)	4 (57%)	3 (17%)	0 (0%)	4 (14%)	-	-	4 (40%)
	2	8 (7%)	3 (8%)	2 (11%)	0 (%)	0 (%)	1 (13%)	2 (7%)	-	-	2 (20%)
	NR	0	0	0	0	0	0	0	-	-	1 (10%)
SARS-CoV-2 infection prior to vaccination		12 (10%)	2 (5%)	3 (16%)	1 (14%)	4 (22%)	1 (13%)	1 (4%)	0 (0%)	-	-
Received steroids within a week of vaccine, n (%)		22 (19%)	0 (0%)	12 (63%)	3 (43%)	7 (39%)	0 (0%)	0 (0%)	-	-	-
Average WCC prior to first vaccination			6.6	9.3	4.9	7.9	6	5.9	-	-	-

Antibody titers after vaccination were evaluated in 117 patients with cancer and 22 HDs, and after infection in 65 unvaccinated patients. T cell responses after vaccination were evaluated in a subset of 66 patients with cancer and 11 HDs.

ECOG PS, Eastern Cooperative Oncology Group performance status; GI, gastrointestinal; GU, genitourinary; Gyn, gynecologic; HD, healthy donors; IMCD, immunocompromised; IO, immunotherapy; NED, no evidence of disease; NR, not reported; Tx, treatment; WCC, white cell count.

### Antibody responses to RBD and Spike

**Figure 1:**
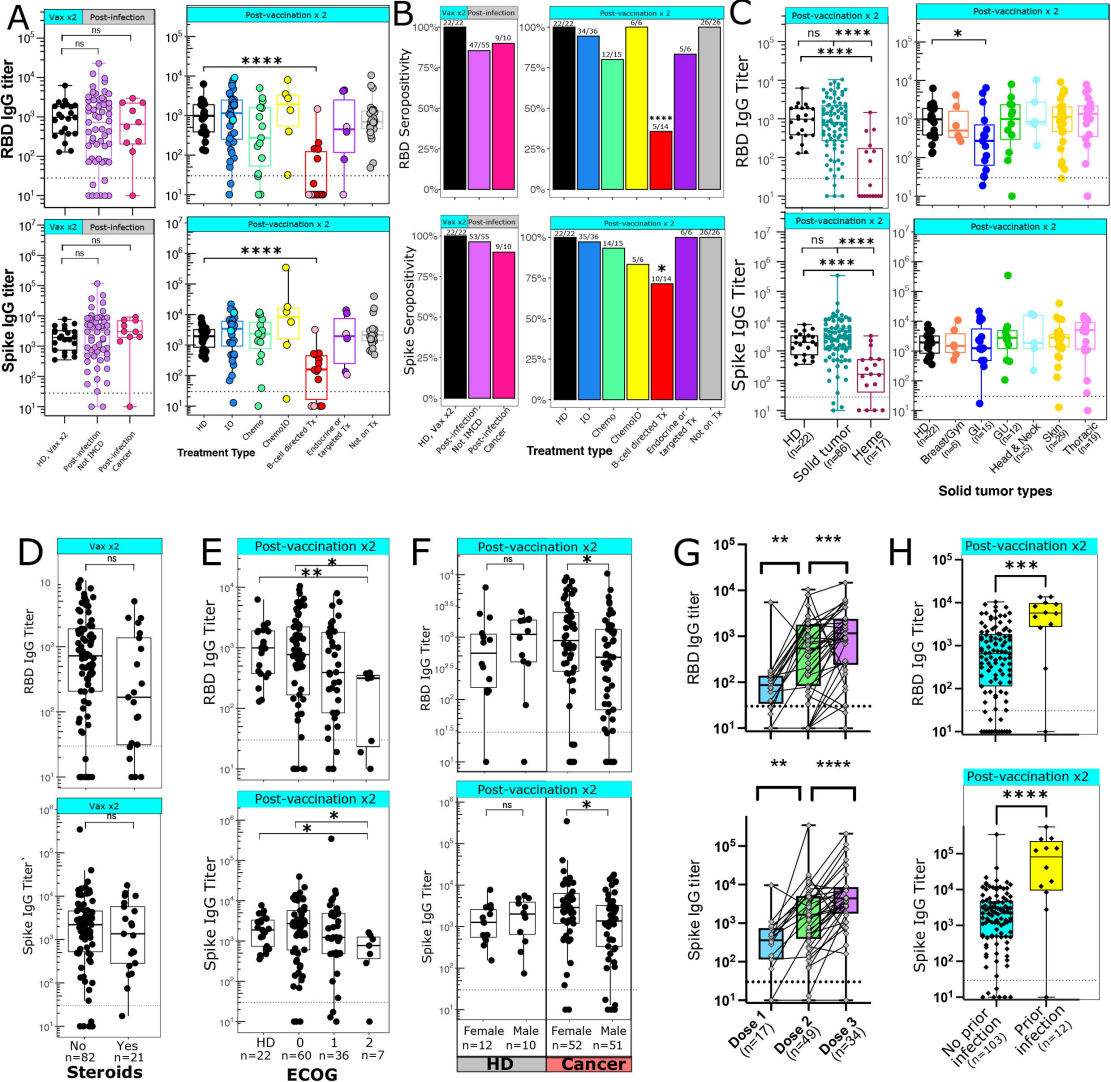
Antibody generation after COVID-19 vaccination. (A) IgG antibody titers against RBD (top panels) and Spike (bottom panels) were measured after infection (left panels) or two vaccine doses (right panels). Dark blue=anti-PD-1 immunotherapies, light blue=non-PD-1 immunotherapies, pink=anti-CD20 antibodies, red=multiple myeloma or other B cell directed therapies, dark purple=targeted therapies, light purple=endocrine therapies. (B) Percentage of patients who are seropositive for RBD (top) or Spike (bottom). Numbers above each bar represent the number of seropositive patients out of the total number of patients per group. Data analyzed using Fisher’s exact test. (C) RBD (top) and spike (bottom) antibody titers after two vaccine doses grouped by cancer types. Solid tumors are broken down into individual tumor subsets in the right panels. (D) Antibody titers after two vaccine doses in patients who did (n=21) or did not (n=82) receive steroids within 1 week of vaccination. (E) Antibody titer after two vaccine doses by ECOG performance status. (F) Antibody titer after two vaccine doses by sex. Left panel shows HDs; right panel shows patients with cancer. (G) Antibody titers after 1, 2, or 3 doses of an mRNA vaccine. Samples from the same patient are connected with a line; data shown is from 55 distinct patients. Data analyzed using Wilcoxon matched-pairs signed rank test. (H) Antibody titers after two vaccine doses in patients with cancer with (n=12) or without (n=103) a documented COVID-19 infection prior to vaccination. Patients with a COVID-19 infection prior to vaccination were excluded from A–G. (A, C–H) Seropositive threshold is indicated with a horizontal dashed line in panels. Boxplots indicated median and 25th and 75th quartiles, with whiskers extending to minimum and maximum. (C–H) Number of patients per group is shown with x-axis label. (A–F, H) Each point shown represents a separate patient. (A, C–F, H) Data analyzed using Wilcoxon rank-sum test. *P≤0.05; **p≤0.01; ***p≤0.001; ****p≤0.0001. chemoIO, chemoimmunotherapy; IO, immunotherapy; ns, not significant; RBD, receptor binding domain; Tx, treatment; ECOG, Eastern Cooperative Oncology Group

Binding IgG antibody levels to the original WT SARS-CoV-2 spike protein and RBD were measured by ELISA after one, two, or three vaccine doses. RBD antibody binding titers correlate with antibody neutralization ability,^7–10^ while Spike antibodies may also include non-neutralizing antibodies and antibodies cross-reactive to other coronavirus strains.^47^ In our cohort, Spike and RBD titers were highly correlated (r=0.92, p<2.2e-16), although some patients who were seronegative for RBD did express low-level but detectable Spike antibodies (online supplemental figure 1A). In HDs, antibody titers after two doses of the vaccine were similar to titers in patients after SARS-CoV-2 infection (figure 1A). We had hypothesized that patients treated with anti-PD-1 immunotherapy may develop higher antibody titers compared with HDs. However, the observed titers and seropositivity rates were similar between both groups (figure 1A, B). One patient in the IO cohort who was seronegative for RBD antibodies was receiving pembrolizumab at the time of vaccination, but had previously received rituximab 8 months prior to vaccination. The other seronegative patient in this group was on first-line pembrolizumab for microsatellite-unstable colon cancer. While there were no significant differences in titers between HDs and patients receiving cytotoxic chemotherapy, there was a trend toward lower RBD antibody levels in patients on chemotherapy as well as a high degree of variability within the group, potentially reflecting the varying degrees of immunosuppression induced by different chemotherapy regimens. In particular, a number of patients with GI malignancies had lower RBD antibody titers, leading to significantly lower median antibody levels in this group (figure 1C). Patients receiving B cell-directed therapies and those with hematological malignancies also had significantly lower titers and seropositivity rates to both RBD and Spike (figure 1A–C). This was seen in both patients receiving anti-CD20 antibodies such as rituximab, as well as proteasome inhibitors such as carfilzomib for multiple myeloma. Patients with cancer who were on endocrine therapies, targeted therapies, or not on active treatment had similar RBD and Spike antibody levels compared with HDs. Overall, 81/86 patients with solid tumors were seropositive for RBD antibodies, and the mean antibody titer was similar among HDs and patients with solid tumors (figure 1C). There was a non-significant trend toward lower RBD IgG antibody levels in patients who received steroids within a week before or after the first or second vaccine (figure 1D). Most of these patients either received dexamethasone 12 mg–20 mg as a premedication before chemotherapy, or 10 mg–40 mg dexamethasone weekly as part of a treatment regimen for multiple myeloma. There was a consistent rate of decline in antibody titers over time observed in six participants who had antibody levels measured at two time points multiple months apart after the second vaccination (online supplemental figure 1B).

10.1136/jitc-2022-004766.supp1 Supplementary data

When looking at demographic factors that may affect antibody titers, we found that patients with an Eastern Cooperative Oncology Group (ECOG) performance status of 2 had lower RBD and Spike antibody levels (figure 1E). Males with cancer had lower RBD and Spike antibody levels compared with females with cancer; no difference was seen in antibody titers between male and female HDs (figure 1F). A modest correlation was seen between age and reduced antibody titer (RBD, R=−0.25, p=0.0044; Spike R=−0.24, p=0.0079) (online supplemental figure 1C). For patients with matched samples after the first, second, and/or third dose of an mRNA vaccine, an increase in RBD and Spike titers was seen after the second and third doses (figure 1G). Six patients who were seronegative after dose 2 had an additional titer drawn after dose 3; 5/6 of these patients became seropositive after a third vaccine dose. Median RBD and Spike antibody titers were higher in vaccinated patients with cancer with a COVID-19 infection prior to vaccination compared with those without a history of prior COVID-19 infection (figure 1H); as a result, these vaccinated patients with prior infections were not included in the other antibody analyses.

After an average follow-up period of 262 days since full vaccination, four oncology patients developed a breakthrough infection at least 14 days after their second vaccine dose. Two patients with a breakthrough infection were on B-cell directed therapies and did not develop RBD or Spike antibodies after the second vaccine dose. The other two patients were on chemotherapy or active surveillance and had RBD titers of 1.7×10^2^ and 5×10^3^, respectively. Three of these breakthrough infections occurred prior to when the omicron variant was documented in the USA in December 2021,^48^ while the breakthrough infection in the patient with the RBD titer of 5×10^3^ occurred in December 2021, 8 months after initial vaccination.

### T cell responses to WT and B.1.617.2 variants

**Figure 2:**
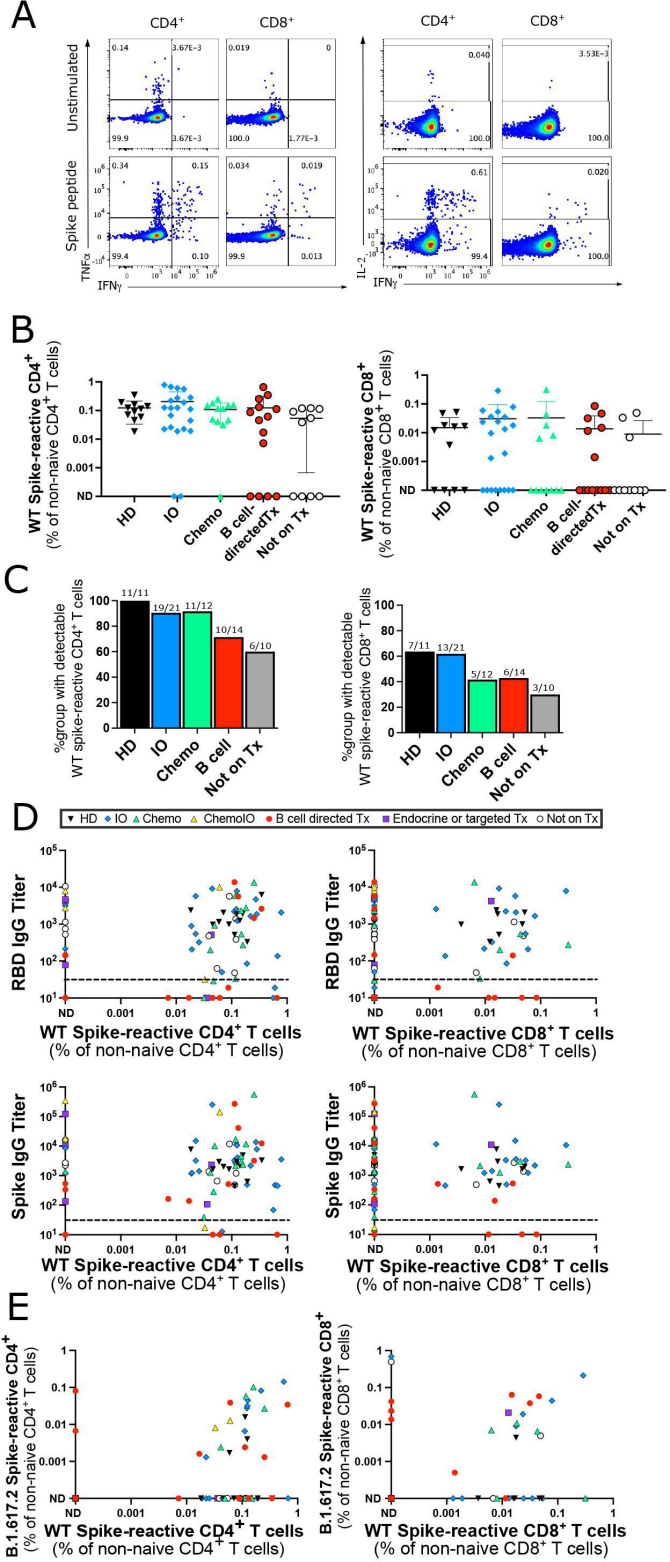
Spike-reactive T cell responses after two vaccine doses. (A) Representative intracellular cytokine staining for IFN-γ, TNF-α, and IL-2 in unstimulated wells (top) and wells stimulated with the WT spike peptide pool (bottom). (B) Percentage of CD4^+^ (left) and CD8^+^ (right) T cells reactive to WT spike peptide pool out of total non-naive CD4^+^ or CD8^+^ T cells. Cells with detectable IFN-γ, TNF-α, or IL-2 after background subtraction of cytokine levels from unstimulated wells were considered peptide-reactive. No significant differences between HD and other groups using Kruskal-Wallis test. (C) Percentage of patients in each group with a detectable CD4^+^ (left) or CD8^+^ (right) T cell response to WT spike peptides after background subtraction of unstimulated wells. Numbers above each bar represent the number of patients with spike-reactive T cells out of the total number of patients per group. No significant differences between HD and other groups using Fisher’s exact test. (D) Correlation between RBD (top graphs) or spike (bottom graphs) IgG antibody titers and percentage of WT spike-reactive CD4^+^ (left graphs) or CD8^+^ (right graphs) T cells out of total CD4^+^ or CD8^+^ non-naive T cells. (E) Correlation of percentage of non-naive CD4^+^ (left) or CD8^+^ (right) cells that react to B.1.617.2 vs WT spike peptide pools. Of note, the B.1.617.2 peptide pool only contains the peptides mutated between the WT and B.1.617.2 variant, but does not include shared peptides. (A–E) All samples from after second vaccination, each point represents a distinct participant (n=77 for WT spike peptides and n=59 for B.1.617.2 spike peptides). (D, E) Treatment type indicated by symbol shape and color in legend. HD, healthy donors; IO, immunotherapy; ND, not detectable; RBD, receptor binding domain; WT, wild-type.

We next investigated the T cell response after 2 doses of an mRNA vaccine in a subset of 11 HDs and 66 patients with cancer. PBMCs were co-cultured with overlapping 15-mer peptide sequences covering the predicted antigenic sequences of the WT spike protein. The 15-mer peptides can bind to both MHC-I and MHC-II molecules and stimulate CD4^+^ and CD8^+^ T cells. Spike-peptide stimulated T cells that produced detectable levels of IFN-γ, TNF-α, or IL-2 by intracellular cytokine staining after subtracting the background level in unstimulated wells were considered to be spike-reactive (figure 2A). No differences in WT spike-peptide T cell reactivity were seen in patients who had a COVID-19 infection prior to vaccination and those who did not (online supplemental figure 2A), so patients with prior COVID-19 infections were included in subsequent T cell analyses. Similar levels of spike-reactive T cells were seen across HDs and oncology patients on IO, chemotherapy, B cell-directed therapies, and those not on treatment (figure 2B, C). Eleven out of thirteen patients tested who were seronegative for antibodies did develop detectable CD4^+^ or CD8^+^ T cell responses against Spike peptides (figure 2D), including 6/8 patients on B cell-directed therapies. T cell responses were still detectable at least 4 months after initial vaccination (online supplemental figure 2B). CD4^+^ T cell responses were more readily detectable compared with CD8^+^ T cell responses in both patients with cancer and HDs (online supplemental figure 2C). CD8^+^ T cell responses against peptides pooled from cytomegalovirus, Epstein-Barr virus, and the influenza virus (CEF) were tested as a positive control; CD8^+^ T cell responses against CEF were detectable in 51/62 samples tested (online supplemental figure 2D).

10.1136/jitc-2022-004766.supp2 Supplementary data

Finally, we investigated whether vaccination with the WT spike protein can induce T cell immunity against the B.1.617.2 (delta) variant of concern. PBMCs were stimulated with a pool of 32 peptides selectively covering only the 10 mutations in the B.1.617.2 variant. Cross-reactive CD4^+^ or CD8^+^ T cells recognizing the B.1.617.2 variant spike peptides were detected in 31/59 samples (figure 2E), suggesting that the T cell response to variants of concern is preserved in many cases.

### Adrenal crisis and irAEs after vaccination

We reviewed medical records to identify significant reactions leading to hospitalization or an ED visit within 24 hours of vaccination. Worsening irAEs within 8 weeks after dose 1 were also reviewed for patients on immune-checkpoint inhibitors. Two patients with pre-existing immune checkpoint inhibitor-induced adrenal insufficiency developed fevers >39.4°C and symptomatic hypoadrenalism after vaccination. One of these patients required ICU admission for hypotension and IV hydrocortisone, while the other patient was successfully treated at home with stress-dose oral hydrocortisone. Of note, this second patient had previously had an adrenal crisis requiring vasopressors after receiving talimogene laherparepvec (T-VEC) therapy 1 year prior to receiving the COVID-19 vaccination. Both patients subsequently developed antibodies (RBD titers 3.4×10^3^ and 5.6×10^3^; Spike titers 3.4×10^3^ and 5.6×10^3^). No other patients had an ED visit or hospitalization with 24 hours of vaccination. A third patient developed autoimmune Type I diabetes about 3 weeks after the second vaccination; this patient had previously developed other irAEs including adrenal insufficiency, transaminitis, and a morbilliform drug rash after receiving ipilimumab and nivolumab. Overall, other than the potential for adrenal crisis in patients with known adrenal insufficiency we did not detect any significant safety concerns after vaccination of patients on anticancer therapies.

## Discussion

In this study, we show that the majority of solid tumor patients develop equivalent antibody and T cell responses after COVID-19 vaccination compared with HDs. Patients on B cell-directed therapy have an impaired humoral immune response but intact T cell response to the COVID-19 vaccine. Although T and B cells express PD-1 and/or PD-L1 and we hypothesized that blockade of the PD-1 pathway may affect antibody generation, we did not see any substantial differences in antibody titer or seropositivity in patients on immune checkpoint inhibitors. While there was more heterogeneity in the magnitude of antibody generation in patients on cytotoxic chemotherapy, 80% of these patients were seropositive for RBD antibodies. Antibody generation was not substantially affected by steroid administration, and only modestly correlated with age. Many patients over age 65, who are at higher risk for severe COVID-19 infection, developed equivalent antibody titers compared with younger patients after vaccination. While patients with hematological malignancies were most likely to be seronegative, a few seronegative patients were detected in all cancer treatment categories. Our results suggest that anti-RBD antibody levels should be routinely checked after COVID-19 vaccination in patients with cancer, and patients who do not mount an antibody response after three doses of the vaccine should receive pre-exposure prophylactic antibody administration.

Our results are consistent with several other recent reports showing substantially impaired antibody generation in patients with hematological malignancies,^49–52^ but largely intact antibody responses in patients on cytotoxic chemotherapy or immunotherapy.^51 53 54^ In some studies, antibody titers or seropositivity rates were mildly lower in patients on chemotherapy^52 55 56^ or chemoimmunotherapy.^57^ Differences in the relative representation of different types of cancer or chemotherapy regimens may explain some of this variation. Interestingly, the CANVAX study demonstrated that patients on immunotherapy had similar spike binding antibody titers but tended to have higher neutralizing titers.^53^ This could suggest that while PD-1 blockade does not affect the size of the antibody response, PD-1 on T_FH_ and/or T_FR_ cells may affect antibody affinity and/or ongoing affinity maturation. A third vaccine dose is now recommended for all adults in the US, and we found that antibody titers increased in 80% of patients after a booster vaccine. Importantly, five of six patients who were seronegative after two doses and had a sample available after the third dose became seropositive after dose 2, consistent with other reports.^58–61^

Our work extends several of these prior studies by also analyzing the T cell response to vaccination. Eighty-five per cent of patients who were seronegative for RBD did develop a detectable CD4^+^ T cell response, consistent with other reports.^58^ In patients on B-cell directed therapy with an impaired humoral immune response during COVID-19 infection, increased levels of CD8^+^ T cells were shown to be protective against severe disease,^62^ suggesting that patients that develop a T cell response but no antibody response after vaccination may still have some protection from severe disease.

Decreased antibody neutralization to both the B.1.617.2 (delta) and B.1.1529 (omicron) variant has been reported,^18 63–66^ so we examined the T cell response to the B.1.617.2 variant. Unlike antibody epitopes which are displayed on the exposed domains of a protein, T cell epitopes can be located throughout the protein^67 68^ and do not depend on the 3-dimensional conformation. After vaccination or infection, most patients develop T cell responses to multiple epitopes across different regions of the spike peptide.^67–69^ This observation suggests that the T cell response is less likely to be affected by amino acid mutations in novel viral variants.^69^ We showed that T cells from 31/59 donors were able to recognize the mutated peptides in the B.1.617.2 variant, despite immunization with a vaccine expressing the WT spike protein. This does not include T cells that would be able to recognize shared epitopes between the WT and B.1.617.2 variants, as the variant peptide pool used only contained the mutated peptides. This may also be an underestimate as this smaller pool of peptides may not efficiently bind to all HLA alleles. Although the true percentage of vaccinated oncology patients with T cells able to recognize viral variants may be higher, our data are consistent with other work showing that many T cells immunized against the WT spike protein are able to recognize viral variants of concern.^69–71^

While the correlated analysis of both the cellular and humoral immune response to vaccination in patients on anticancer therapies is a strength of this study, there are some limitations. Our HD cohort tended to be younger than the patients with cancer. We measured RBD binding antibody titers, which strongly correlate with neutralizing antibody levels^7–10^ but may not precisely reflect neutralization ability. We measured T cell responses to a megapool of peptides from the spike protein. This could include some pre-existing T cells cross-reactive to spike proteins from the common cold coronaviruses^69 72 73^ rather than vaccine-induced spike-reactive T cells, but any cross-reactive cells identified by these assays would also be expected to be active against a potential SARS-CoV-2 infection. There may also be low-frequency spike-reactive T cells in some patients that are below the limit of detection for intracellular cytokine staining, particularly at later time points after the contraction of the immune response to vaccination. This was a real-world study with samples collected during standard of care oncology visits, so dose 2 samples were collected on average 55 days after the second dose, with 79% of samples collected within 90 days of the second vaccination. While this heterogeneity in timing may have missed peak antibody titers and/or maximal T cell responses in some patients, half-lives of up to 116 days for RBD antibodies and 207 days for T cell responses have been observed.^14 16^ This study was conducted prior to the rise of the omicron variant, and future work will be needed to fully assess antibody and T cell responses to omicron in patients with cancer.

Our work has several important clinical implications. Routine antibody testing post-vaccination is not currently recommended by the Centers for Disease Control and Prevention (CDC) or the Food and Drug Administration (FDA),^74^ as there are not well-defined guidelines for interpreting how antibody titers correlate with protection from breakthrough infections or severe disease. Additionally, as we have shown here, many patients without a detectable antibody response still have a T cell response which may provide some protection against severe disease.^62^ However, we argue that post-vaccination antibody testing could be useful in oncology patients to help identify immunocompromised patients who might receive the most benefit from prophylactic antibody administration. Tixagevimab packaged with cilgavimab (Evusheld) has received emergency use approval for pre-exposure prophylaxis in moderately and severely immunocompromised patients who may not mount an adequate immune response to vaccination.^75 76^ This includes patients receiving active treatment for solid tumors and hematological malignancies. Measuring post-vaccination antibody titers is not recommended or required prior to administration, which is important to avoid operational or logistical obstacles to administration. Severely immunocompromised patients, including post-transplant patients and patients receiving B cell-depleting therapies, are least likely to mount an adequate antibody response to COVID-19 vaccination and are being prioritized for pre-exposure prophylaxis while supplies are limited.^75^ The antibody response post-vaccination is more variable in patients being treated for solid tumors, who are considered moderately immunocompromised. While patients with solid tumors who want pre-exposure prophylaxis should continue to receive it without requiring antibody testing, knowledge of a patient’s antibody levels may help inform an individualized conversation about the potential benefits of tixagevimab and cilgavimab for patients who are undecided. Immunocompromised patients who do not have detectable spike or RBD antibodies at least 1 week after the second or third vaccination, when antibody levels are expected to peak,^10^ are likely to benefit from pre-exposure prophylaxis. Of note, antibody tests against the nucleocapsid protein indicate prior infection and should not be used to determine if a patient has mounted an immune response against vaccines expressing the spike protein.^74^ Several models^12 13^ have been developed to predict the correlation between antibody titers and vaccine efficacy, and further validation of this relationship is needed to aid in the development of clinical guidelines for interpreting antibody testing in immunocompromised patients.

Overall, COVID-19 vaccinations have been shown to be safe in patients with cancer. Our experience highlights the importance of reminding patients with known adrenal insufficiency to have a low threshold to take stress dose steroids at the first signs of an adrenal crisis after vaccination. Vaccine antigens and adjuvants are recognized by the innate immune system as damage-associated molecular patterns, leading to cytokine production, fevers, and systemic symptoms similar to those seen during mild infections.^77^ In addition to COVID-19 vaccines, other reactogenic vaccines such as the recombinant zoster vaccine (Shingrix) which have a high incidence of fever post-vaccination^78^ may also cause increased levels of physiological stress warranting stress-dose steroids for patients with adrenal insufficiency. Our data and others have shown that steroid administration around the time of vaccination does not substantially suppress the antibody response.^51 54^ The only new irAE seen after COVID-19 vaccination was in a patient with a history of three other irAEs which developed prior to vaccination, and therefore, it is unlikely that the new irAE was related specifically to vaccination. Our results add to the literature demonstrating the safety of COVID-19 and influenza vaccines among patients on immunotherapy.^54 59 79–81^ Our results also add to the growing body of literature demonstrating that most patients on anticancer therapeutics will make an antibody and/or T cell response to COVID-19 vaccination, and should be encouraged to receive three doses of an mRNA-based vaccine as soon as possible even while on active anticancer therapies.

## Data Availability

All data relevant to the study are included in the article or uploaded as online supplemental information.

## References

[1] Polack FP, Thomas SJ, Kitchin N, et al. Safety and efficacy of the BNT162b2 mRNA Covid-19 vaccine. N Engl J Med 2020;383:2603–15. 10.1056/NEJMoa2034577 33301246 PMC7745181

[2] Baden LR, El Sahly HM, Essink B, et al. Efficacy and safety of the mRNA-1273 SARS-CoV-2 vaccine. N Engl J Med 2021;384:403–16. 10.1056/NEJMoa2035389 33378609 PMC7787219

[3] Sadoff J, Gray G, Vandebosch A, et al. Safety and efficacy of single-dose Ad26.COV2.S vaccine against Covid-19. N Engl J Med 2021;384:2187–201. 10.1056/NEJMoa2101544 33882225 PMC8220996

[4] Kuderer NM, Choueiri TK, Shah DP, et al. Clinical impact of COVID-19 on patients with cancer (CCC19): a cohort study. Lancet 2020;395:1907–18. 10.1016/S0140-6736(20)31187-9 32473681 PMC7255743

[5] Garassino MC, Whisenant JG, Huang L-C, et al. COVID-19 in patients with thoracic malignancies (TERAVOLT): first results of an international, registry-based, cohort study. Lancet Oncol 2020;21:914–22. 10.1016/S1470-2045(20)30314-4 32539942 PMC7292610

[6] Piccoli L, Park Y-J, Tortorici MA, et al. Mapping neutralizing and immunodominant sites on the SARS-CoV-2 spike receptor-binding domain by structure-guided high-resolution serology. Cell 2020;183:1024–42. 10.1016/j.cell.2020.09.037 32991844 PMC7494283

[7] Suthar MS, Zimmerman MG, Kauffman RC, et al. Rapid generation of neutralizing antibody responses in COVID-19 patients. Cell Rep Med 2020;1:100040. 10.1016/j.xcrm.2020.100040 32835303 PMC7276302

[8] Jackson LA, Anderson EJ, Rouphael NG, et al. An mRNA Vaccine against SARS-CoV-2 - Preliminary Report. N Engl J Med 2020;383:1920–31. 10.1056/NEJMoa2022483 32663912 PMC7377258

[9] Sahin U, Muik A, Derhovanessian E, et al. COVID-19 vaccine BNT162b1 elicits human antibody and TH1 T cell responses. Nature 2020;586:594–9. 10.1038/s41586-020-2814-7 32998157

[10] Goel RR, Painter MM, Apostolidis SA, et al. mRNA vaccines induce durable immune memory to SARS-CoV-2 and variants of concern. Science 2021;374:eabm0829. 10.1126/science.abm0829 PMC9284784 34648302

[11] Gilbert PB, Montefiori DC, McDermott AB, et al. Immune correlates analysis of the mRNA-1273 COVID-19 vaccine efficacy clinical trial. Science 2022;375:43–50. 10.1126/science.abm3425 34812653 PMC9017870

[12] Khoury DS, Cromer D, Reynaldi A, et al. Neutralizing antibody levels are highly predictive of immune protection from symptomatic SARS-CoV-2 infection. Nat Med 2021;27:1205–11. 10.1038/s41591-021-01377-8 34002089

[13] Earle KA, Ambrosino DM, Fiore-Gartland A, et al. Evidence for antibody as a protective correlate for COVID-19 vaccines. Vaccine 2021;39:4423–8. 10.1016/j.vaccine.2021.05.063 34210573 PMC8142841

[14] Doria-Rose N, Suthar MS, Makowski M, et al. Antibody persistence through 6 months after the second dose of mRNA-1273 vaccine for Covid-19. N Engl J Med 2021;384:2259–61. 10.1056/NEJMc2103916 33822494 PMC8524784

[15] Mateus J, Dan JM, Zhang Z, et al. Low-dose mRNA-1273 COVID-19 vaccine generates durable memory enhanced by cross-reactive T cells. Science 2021;374:eabj9853. 10.1126/science.abj9853 34519540 PMC8542617

[16] Dan JM, Mateus J, Kato Y, et al. Immunological memory to SARS-CoV-2 assessed for up to 8 months after infection. Science 2021;371:4063. 10.1126/science.abf4063 PMC7919858 33408181

[17] Cohen KW, Linderman SL, Moodie Z, et al. Longitudinal analysis shows durable and broad immune memory after SARS-CoV-2 infection with persisting antibody responses and memory B and T cells. Cell Rep Med 2021;2:100354. 10.1016/j.xcrm.2021.100354 34250512 PMC8253687

[18] Garcia-Beltran WF, St Denis KJ, Hoelzemer A, et al. mRNA-based COVID-19 vaccine boosters induce neutralizing immunity against SARS-CoV-2 omicron variant. Cell 2022;185:457–66. 10.1016/j.cell.2021.12.033 34995482 PMC8733787

[19] Pauken KE, Godec J, Odorizzi PM, et al. The PD-1 Pathway Regulates Development and Function of Memory CD8^+^ T Cells following Respiratory Viral Infection. Cell Rep 2020;31:107827. 10.1016/j.celrep.2020.107827 32610128 PMC7377452

[20] Ahn E, Araki K, Hashimoto M, et al. Role of PD-1 during effector CD8 T cell differentiation. Proc Natl Acad Sci U S A 2018;115:4749–54. 10.1073/pnas.1718217115 29654146 PMC5939075

[21] Verma V, Shrimali RK, Ahmad S, et al. PD-1 blockade in subprimed CD8 cells induces dysfunctional PD-1^+^CD38^hi^ cells and anti-PD-1 resistance. Nat Immunol 2019;20:1231–43. 10.1038/s41590-019-0441-y 31358999 PMC7472661

[22] Thibult M-L, Mamessier E, Gertner-Dardenne J, et al. PD-1 is a novel regulator of human B-cell activation. Int Immunol 2013;25:129–37. 10.1093/intimm/dxs098 23087177

[23] Nishimura H, Minato N, Nakano T, et al. Immunological studies on PD-1 deficient mice: implication of PD-1 as a negative regulator for B cell responses. Int Immunol 1998;10:1563–72. 10.1093/intimm/10.10.1563 9796923

[24] Good-Jacobson KL, Szumilas CG, Chen L, et al. PD-1 regulates germinal center B cell survival and the formation and affinity of long-lived plasma cells. Nat Immunol 2010;11:535–42. 10.1038/ni.1877 20453843 PMC2874069

[25] Hamel KM, Cao Y, Wang Y, et al. B7-H1 expression on non-B and non-T cells promotes distinct effects on T- and B-cell responses in autoimmune arthritis. Eur J Immunol 2010;40:3117–27. 10.1002/eji.201040690 21061440 PMC3638795

[26] Crotty S. T follicular helper cell biology: a decade of discovery and diseases. Immunity 2019;50:1132–48. 10.1016/j.immuni.2019.04.011 31117010 PMC6532429

[27] Khan AR, Hams E, Floudas A, et al. PD-L1hi B cells are critical regulators of humoral immunity. Nat Commun 2015;6:5997. 10.1038/ncomms6997 25609381

[28] Hams E, McCarron MJ, Amu S, et al. Blockade of B7-H1 (programmed death ligand 1) enhances humoral immunity by positively regulating the generation of T follicular helper cells. J Immunol 2011;186:5648–55. 10.4049/jimmunol.1003161 21490158

[29] Gajewski T, Rouhani S, Trujillo J, et al. Severe COVID-19 infection is associated with aberrant cytokine production by infected lung epithelial cells rather than by systemic immune dysfunction. Res Sq 2021:21266492. 10.21203/rs.3.rs-1083825/v1

[30] Amanat F, Stadlbauer D, Strohmeier S, et al. A serological assay to detect SARS-CoV-2 seroconversion in humans. Nat Med 2020;26:1033–6. 10.1038/s41591-020-0913-5 32398876 PMC8183627

[31] Ritz C, Baty F, Streibig JC, et al. Dose-response analysis using R. PLoS One 2015;10:e0146021. 10.1371/journal.pone.0146021 26717316 PMC4696819

[32] Aphalo PJ. ggpmisc: miscellaneous extensions to ‘ggplot2’ 2021.

[33] Kassambara A. ggpubr: ‘ggplot2’ based publication ready plots. (Github).

[34] Ahlmann-Eltze C. ggsignif: significance brackets for ‘ggplot2’ 2019.

[35] Auguie B, Antonov A. gridExtra: miscellaneous functions for ‘grid’ graphics. R package version 2 2017.

[36] Harrell FE, Dupont C. Hmisc: Harrell miscellaneous 2020.

[37] Firke S. Janitor: simple tools for examining and cleaning dirty data. R package version 2 2020.

[38] Grolemund G, Wickham H. Dates and times made easy with lubridate. J Stat Softw 2011;40:1–25. 10.18637/jss.v040.i03

[39] Bache SM, Wickham H. magrittr: a forward-pipe operator for R 2020.

[40] Pedersen TL. patchwork: the composer of plots 2020.

[41] Wickham H, Bryan J. readxl: read excel files 2019.

[42] Wickham H. Reshaping data with the reshape package. J Stat Softw 2007;21:1–20. 10.18637/jss.v021.i12

[43] Wickham H, Seidel D. scales: scale functions for visualization 2020.

[44] Wickham H. stringr: simple, consistent wrappers for common string operations 2019.

[45] Wickham H, Averick M, Bryan J, et al. Welcome to the tidyverse. J Open Source Softw 2019;4:1686. 10.21105/joss.01686

[46] Ooms J. writexl: export data frames to Excel ‘xlsx’ format 2020.

[47] Anderson EM, Goodwin EC, Verma A, et al. Seasonal human coronavirus antibodies are boosted upon SARS-CoV-2 infection but not associated with protection. Cell 2021;184:1858–64. 10.1016/j.cell.2021.02.010 33631096 PMC7871851

[48] First confirmed case of omicron variant detected in the United States, 2021. Available: https://www.cdc.gov/media/releases/2021/s1201-omicron-variant.html

[49] Terpos E, Gavriatopoulou M, Ntanasis-Stathopoulos I, et al. The neutralizing antibody response post COVID-19 vaccination in patients with myeloma is highly dependent on the type of anti-myeloma treatment. Blood Cancer J 2021;11:138. 10.1038/s41408-021-00530-3 34341335 PMC8327056

[50] Bagacean C, Letestu R, Al-Nawakil C, et al. Humoral response to mRNA anti-COVID-19 vaccines BNT162b2 and mRNA-1273 in patients with chronic lymphocytic leukemia. Blood Adv 2022;6:207–11. 10.1182/bloodadvances.2021006215 34844264 PMC8632355

[51] Thakkar A, Gonzalez-Lugo JD, Goradia N, et al. Seroconversion rates following COVID-19 vaccination among patients with cancer. Cancer Cell 2021;39:1081–90. 10.1016/j.ccell.2021.06.002 34133951 PMC8179248

[52] Addeo A, Shah PK, Bordry N, et al. Immunogenicity of SARS-CoV-2 messenger RNA vaccines in patients with cancer. Cancer Cell 2021;39:1091–8. 10.1016/j.ccell.2021.06.009 34214473 PMC8218532

[53] Naranbhai V, Pernat CA, Gavralidis A, et al. Immunogenicity and reactogenicity of SARS-CoV-2 vaccines in patients with cancer: the CANVAX cohort study. J Clin Oncol 2022;40:2101891. 10.1200/JCO.21.01891 PMC8683230 34752147

[54] Oosting SF, van der Veldt AAM, GeurtsvanKessel CH, et al. mRNA-1273 COVID-19 vaccination in patients receiving chemotherapy, immunotherapy, or chemoimmunotherapy for solid tumours: a prospective, multicentre, non-inferiority trial. Lancet Oncol 2021;22:1681–91. 10.1016/S1470-2045(21)00574-X 34767759 PMC8577843

[55] Grinshpun A, Rottenberg Y, Ben-Dov IZ, et al. Serologic response to COVID-19 infection and/or vaccine in cancer patients on active treatment. ESMO Open 2021;6:100283. 10.1016/j.esmoop.2021.100283 34634634 PMC8469519

[56] Goshen-Lago T, Waldhorn I, Holland R, et al. Serologic status and toxic effects of the SARS-CoV-2 BNT162b2 vaccine in patients undergoing treatment for cancer. JAMA Oncol 2021;7:1507–13. 10.1001/jamaoncol.2021.2675 34236381 PMC8267843

[57] Massarweh A, Eliakim-Raz N, Stemmer A, et al. Evaluation of seropositivity following BNT162b2 messenger RNA vaccination for SARS-CoV-2 in patients undergoing treatment for cancer. JAMA Oncol 2021;7:1133–40. 10.1001/jamaoncol.2021.2155 34047765 PMC8164144

[58] Shroff RT, Chalasani P, Wei R, et al. Immune responses to two and three doses of the BNT162b2 mRNA vaccine in adults with solid tumors. Nat Med 2021;27:2002–11. 10.1038/s41591-021-01542-z 34594036 PMC9004706

[59] Ligumsky H, Dor H, Etan T, et al. Immunogenicity and safety of BNT162b2 mRNA vaccine booster in actively treated patients with cancer. Lancet Oncol 2022;23:193-195. 10.1016/S1470-2045(21)00715-4 34953523 PMC8700280

[60] Gounant V, Ferré VM, Soussi G, et al. Efficacy of severe acute respiratory syndrome coronavirus-2 vaccine in patients with thoracic cancer: a prospective study supporting a third dose in patients with minimal serologic response after two vaccine doses. J Thorac Oncol 2022;17:239–51. 10.1016/j.jtho.2021.10.015 34798306 PMC8593625

[61] Fenioux C, Teixeira L, Fourati S, et al. SARS-CoV-2 antibody response to 2 or 3 doses of the BNT162b2 vaccine in patients treated with anticancer agents. JAMA Oncol 2022;8:612-617. 10.1001/jamaoncol.2021.7777 34994776 PMC8742219

[62] Bange EM, Han NA, Wileyto P, et al. CD8^+^ T cells contribute to survival in patients with COVID-19 and hematologic cancer. Nat Med 2021;27:1280–9. 10.1038/s41591-021-01386-7 34017137 PMC8291091

[63] Planas D, Saunders N, Maes P, et al. Considerable escape of SARS-CoV-2 omicron to antibody neutralization. Nature 2022;602:671–5. 10.1038/s41586-021-04389-z 35016199

[64] Planas D, Veyer D, Baidaliuk A, et al. Reduced sensitivity of SARS-CoV-2 variant delta to antibody neutralization. Nature 2021;596:276–80. 10.1038/s41586-021-03777-9 34237773

[65] Edara V-V, Pinsky BA, Suthar MS, et al. Infection and vaccine-induced neutralizing-antibody responses to the SARS-CoV-2 B.1.617 variants. N Engl J Med 2021;385:664–6. 10.1056/NEJMc2107799 34233096 PMC8279090

[66] Valanparambil R, Carlisle J, Linderman S, et al. Antibody response to SARS-CoV-2 mRNA vaccine in lung cancer patients: reactivity to vaccine antigen and variants of concern. medRxiv 2022:22268599. 10.1101/2022.01.03.22268599

[67] Tarke A, Sidney J, Kidd CK, et al. Comprehensive analysis of T cell immunodominance and immunoprevalence of SARS-CoV-2 epitopes in COVID-19 cases. Cell Rep Med 2021;2:100204. 10.1016/j.xcrm.2021.100204 33521695 PMC7837622

[68] Angyal A, Longet S, Moore SC, et al. T-cell and antibody responses to first BNT162b2 vaccine dose in previously infected and SARS-CoV-2-naive UK health-care workers: a multicentre prospective cohort study. Lancet Microbe 2022;3:e21–31. 10.1016/S2666-5247(21)00275-5 34778853 PMC8577846

[69] Woldemeskel BA, Garliss CC, Blankson JN. SARS-CoV-2 mRNA vaccines induce broad CD4+ T cell responses that recognize SARS-CoV-2 variants and HCoV-NL63. J Clin Invest 2021;131:149335. 10.1172/JCI149335 33822770 PMC8121504

[70] Tarke A, Sidney J, Methot N, et al. Impact of SARS-CoV-2 variants on the total CD4^+^ and CD8^+^ T cell reactivity in infected or vaccinated individuals. Cell Rep Med 2021;2:100355. 10.1016/j.xcrm.2021.100355 34230917 PMC8249675

[71] GeurtsvanKessel CH et al. Divergent SARS CoV-2 Omicron-specific T- and B-cell responses in COVID-19 vaccine recipients. medRxiv 2021:21268416. 10.1126/sciimmunol.abo2202 PMC8939771 35113647

[72] Mateus J, Grifoni A, Tarke A, et al. Selective and cross-reactive SARS-CoV-2 T cell epitopes in unexposed humans. Science 2020;370:89–94. 10.1126/science.abd3871 32753554 PMC7574914

[73] Grifoni A, Weiskopf D, Ramirez SI, et al. Targets of T cell responses to SARS-CoV-2 coronavirus in humans with COVID-19 disease and unexposed individuals. Cell 2020;181:1489–501. 10.1016/j.cell.2020.05.015 32473127 PMC7237901

[74] Centers for Disease Control and Prevention. Interim guidelines for COVID-19 antibody testing, 2022. Available: https://www.cdc.gov/coronavirus/2019-ncov/lab/resources/antibody-tests-guidelines.html

[75] COVID-19 Treatment Guidelines Panel. COVID-19 treatment guidelines: prioritization of anti-SARS-CoV-2 therapies for the treatment and prevention of COVID-19 when there are logistical or supply constraints, 2022. Available: https://www.covid19treatmentguidelines.nih.gov/overview/prioritization-of-therapeutics/

[76] Food and Drug Administration. Fact sheet for healthcare providers: emergency use authorization for EvusheldTM (tixagevimab co-packaged with cilgavimab), 2022. Available: https://www.fda.gov/media/154701/download

[77] Hervé C, Laupèze B, Del Giudice G, et al. The how’s and what’s of vaccine reactogenicity. NPJ Vaccines 2019;4:39. 10.1038/s41541-019-0132-6 31583123 PMC6760227

[78] Lal H, Cunningham AL, Godeaux O, et al. Efficacy of an adjuvanted herpes zoster subunit vaccine in older adults. N Engl J Med 2015;372:2087–96. 10.1056/NEJMoa1501184 25916341

[79] Waissengrin B, Agbarya A, Safadi E, et al. Short-term safety of the BNT162b2 mRNA COVID-19 vaccine in patients with cancer treated with immune checkpoint inhibitors. Lancet Oncol 2021;22:581–3. 10.1016/S1470-2045(21)00155-8 33812495 PMC8016402

[80] Wijn DH, Groeneveld GH, Vollaard AM, et al. Influenza vaccination in patients with lung cancer receiving anti-programmed death receptor 1 immunotherapy does not induce immune-related adverse events. Eur J Cancer 2018;104:182–7. 10.1016/j.ejca.2018.09.012 30368069

[81] Failing JJ, Ho TP, Yadav S, et al. Safety of influenza vaccine in patients with cancer receiving pembrolizumab. JCO Oncol Pract 2020;16:e573–80. 10.1200/JOP.19.00495 32048920 PMC8462591

