# Supplementary material for: Antibody and T cell responses to COVID-19 vaccination in patients receiving anticancer therapies
Source: J Immunother Cancer. 2022 Jun 22;10(6):e004766. doi: 10.1136/jitc-2022-004766 (PMC9226983; doi:10.1136/jitc-2022-004766)
Supplement: Supplementary data [file jitc-2022-004766supp001.pdf]

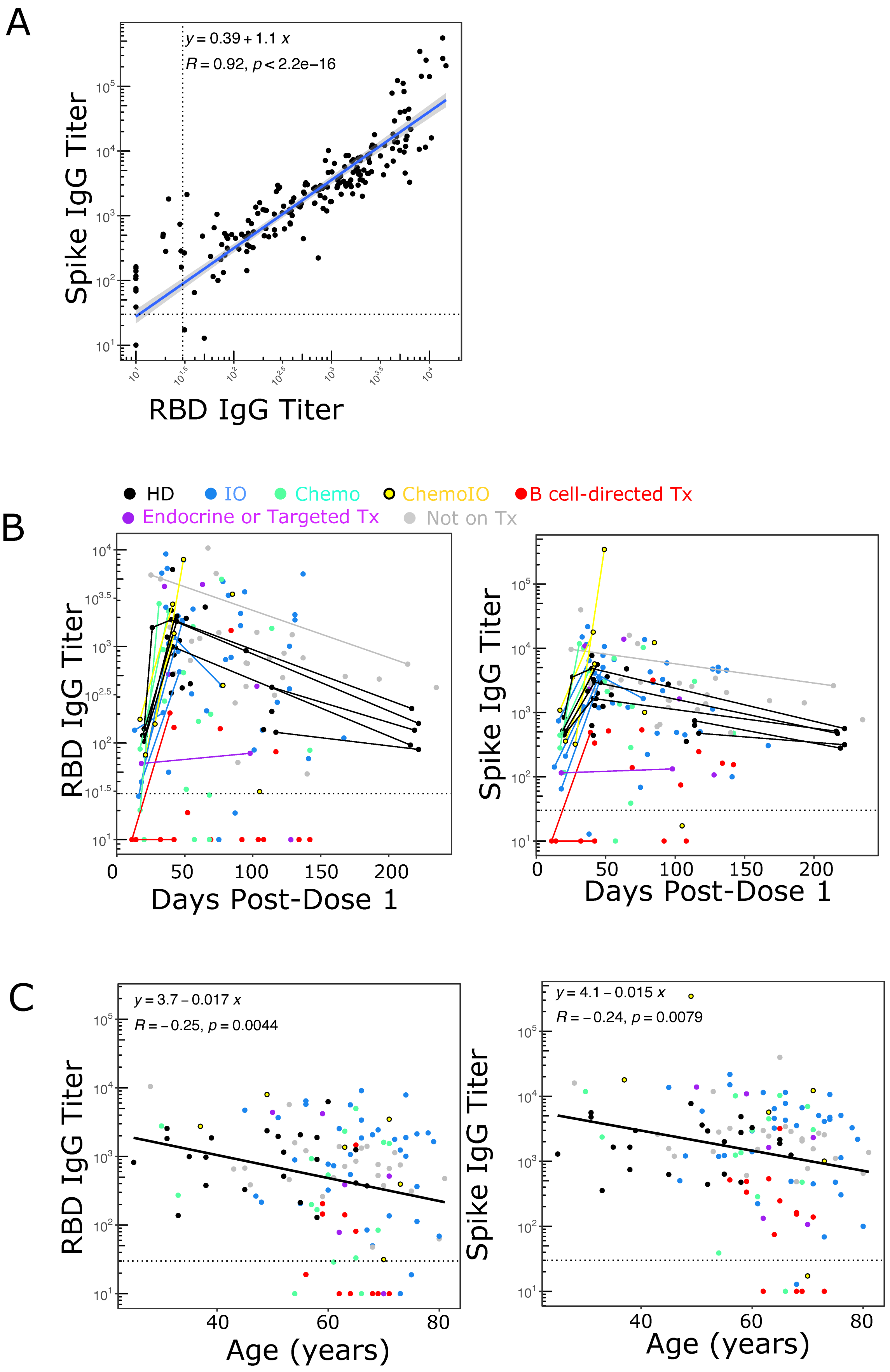

Supplemental Fig 1: (A) Correlation between RBD and spike IgG antibody titers after vaccination. Linear regression was performed on log-transformed RBD and spike titer values. Graph includes all timepoints and includes both vaccinated healthy donors and vaccinated patients with cancer. Vaccinated patients who also had a prior COVID-19 infection are included. (B) Kinetics of RBD (left) and spike (right) antibody titers over time since first vaccination. Multiple samples from the same patient are connected with a line. Samples after dose 1 and after dose 2 are included, but samples after dose 3 are excluded. (C) Linear regression was performed to model the effects of age on the log-transformed RBD (left) and Spike (right) IgG titer values after second vaccination. (A,C) Correlation coefficient (r), p-values, and linear regression are shown in figure. (B - C) Treatment category is indicated by dot color as indicated in legend.

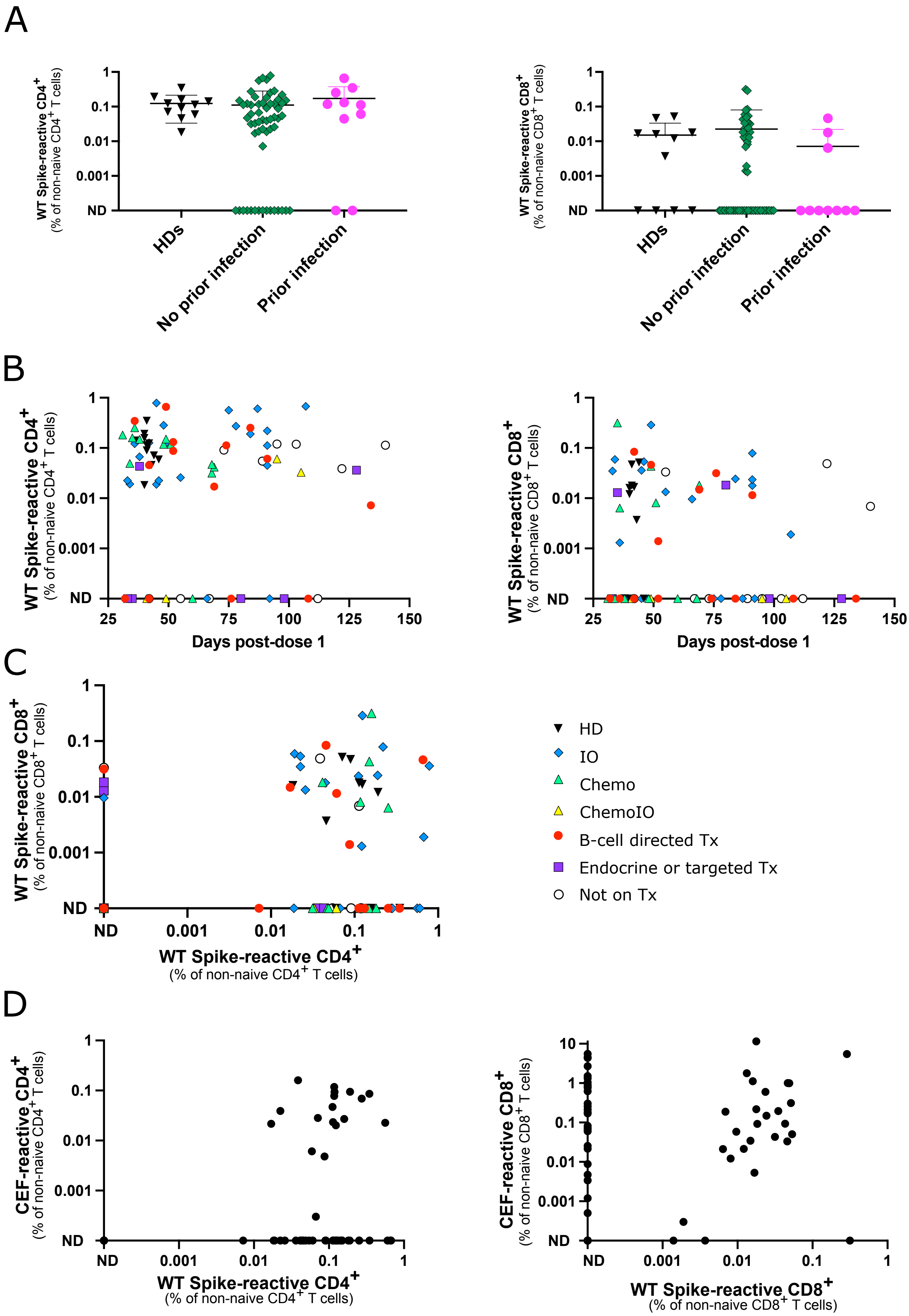

Supplemental Fig 2: (A) Percentage of WT spike-reactive CD4+ (left) or CD8+ (right) out of total non-naive CD4+ or CD8+ T cells after 2 vaccine doses in HDs (n=11) and patients with cancer with (n=10) or without (n=57) a documented COVID-19 infection prior to vaccination. (B) Percentage of WT spike-reactive CD4+ (left) or CD8+ (right) out of total non-naive CD4+ or CD8+ T cells after 2 vaccine doses over time since initial vaccination; n = 78. (C) Comparison between CD4+ and CD8+ WT spike-reactive T cells; n = 78. (D) Comparison between WT-spike and CEF-reactive CD4+ (left) or CD8+ (right) T cells; n = 62. (A-D) All graphs show T cell-responses after 2 vaccine doses and each point represents a distinct participant. (B-C) Treatment type indicated by symbol shape and color in legend. ND = not detectable after background subtraction.

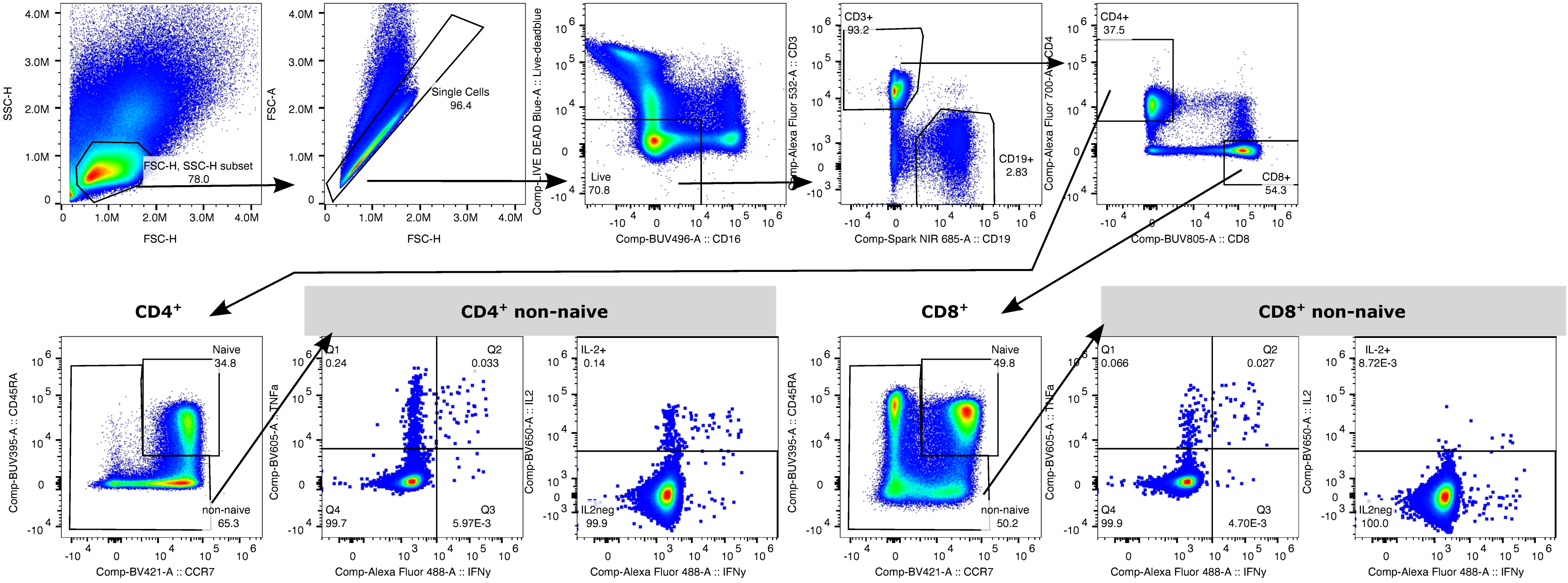

Supplemental Figure 3: Gating strategy for flow-cytometry data.
